# Analyzing the Blueprint: Exploring the Molecular Profile of Metastasis and Therapeutic Resistance

**DOI:** 10.3390/ijms26146954

**Published:** 2025-07-20

**Authors:** Guadalupe Avalos-Navarro, Martha Patricia Gallegos-Arreola, Emmanuel Reyes-Uribe, Luis Felipe Jave Suárez, Gildardo Rivera-Sánchez, Héctor Rangel-Villalobos, Ana Luisa Madriz-Elisondo, Itzae Adonai Gutiérrez Hurtado, Juan José Varela-Hernández, Ramiro Ramírez-Patiño

**Affiliations:** 1 Departamento de Ciencias Médicas y de la Vida, Centro Universitario de la Ciénega (CUCIÉNEGA), Universidad de Guadalajara, Av. Universidad 1115, Lindavista, Ocotlán 47820, Mexico; guadalupe.avalos5337@academicos.udg.mx (G.A.-N.); emmanuel.reyes@academicos.udg.mx (E.R.-U.); hector.rangel@academicos.udg.mx (H.R.-V.); ana.madriz@academicos.udg.mx (A.L.M.-E.); juan.varela@academicos.udg.mx (J.J.V.-H.); 2 División de Genética, Centro de Investigación Biomédica de Occidente (CIBO), Instituto Mexicano del Seguro Social (IMSS), Sierra Mojada 800, Independencia Oriente, Guadalajara 44340, Mexico; marthapatriciagallegos08@gmail.com; 3 División de Inmunología, Centro de Investigación Biomédica de Occidente (CIBO), Instituto Mexicano del Seguro Social (IMSS), Guadalajara 44340, Mexico; luis.jave@academicos.udg.mx; 4 Laboratorio de Biotecnología Farmacéutica, Centro de Biotecnología Genómica, Instituto Politécnico Nacional, Reynosa 88710, Mexico; gildardors@hotmail.com; 5 Departamento de Biología Molecular y Genómica, Centro Universitario de Ciencias de la Salud, Guadalajara 44340, Mexico; itzae.gutierrez@academicos.udg.mx

**Keywords:** cancer, immunity, drug resistance, treatment, drugs, genetics, genomics, metabolism

## Abstract

Metastases are the leading cause of cancer-related deaths. The spread of neoplasms involves multiple mechanisms, with metastatic tumors exhibiting molecular behaviors distinct from their primary counterparts. The key hallmarks of metastatic lesions include chromosomal instability, copy number alterations (CNAs), and a reduced degree of subclonality. Furthermore, metabolic adaptations such as enhanced glycogen synthesis and storage, as well as increased fatty acid oxidation (FAO), play a critical role in sustaining energy supply in metastases and contributing to chemoresistance. FAO promotes the infiltration of macrophages into the tumor, where they polarize to the M2 phenotype, which is associated with immune suppression and tissue remodeling. Additionally, the tumor microbiome and the action of cytotoxic drugs trigger neutrophil extravasation through inflammatory pathways. Chemoresistant neutrophils in the tumor microenvironment can suppress effector lymphocyte activation and facilitate the formation of neutrophil extracellular traps (NETs), which are linked to drug resistance. This article examines the genomic features of metastatic tumors, along with the metabolic and immunological dynamics within the metastatic tumor microenvironment, and their contribution to drug resistance. It also discusses the molecular mechanisms underlying resistance to chemotherapeutic agents commonly used in the treatment of metastatic cancer.

## 1. Introduction

Cancer represents a significant global public health issue, with over 90% of cancer-related deaths attributed to metastasis [1,2,3]. At this advanced stage, tumor cells undergo profound changes, acquiring new characteristics that provide them with a selective advantage and resistance to apoptosis [4,5]. These alterations arise from chromosomal instability, alongside other genetic, metabolic, and molecular shifts that occur as tumors propagate [6,7]. Previous research has demonstrated that the molecular profile of metastatic tumors differs significantly from that of the primary tumor [6], and these differences may be linked to chemoresistance. During cancer progression, the transition to a metastatic phenotype enables cells to acquire mesenchymal and evolutionary traits, rendering them less susceptible to cytotoxic treatments [8]. While some studies suggest that metastases exhibit a lower degree of subclonality, other findings highlight chromosomal aberrations and specific copy number alterations (CNAs) in tumor suppressor genes (TGS) that enable metastatic cells to evade apoptosis [9,10,11]. Furthermore, metastatic cells exhibit remarkable metabolic flexibility, allowing them to adapt to a range of conditions [12]. This metabolic reprogramming enables tumor cells to utilize non-glucosidic substrates, such as glutamine and fatty acid oxidation (FAO), to generate energy, an essential factor for survival and growth in distant tissues [13,14]. This capacity to adjust to nutrient scarcity, hypoxia, and other hostile conditions found in metastatic sites is a key factor in their persistence and resistance to therapies [15], and at the same time, this metabolic reprogramming in tumors contributes to chemoresistance. Specifically, enzymes such as CPT1 (carnitine palmitoyltransferase I) and ACSL4 (long-chain fatty acyl synthetase 4), which are involved in lipid metabolism, show altered expression. These enzymes help integrate lipids and other substrates into cancer cell metabolism, making them resilient to chemotherapy [16,17]. Concerning the tumor microenvironment, immune modulation, as well as tumor-associated neutrophils and the other cells of innate immunity play a crucial role in shaping the microenvironment. Their activity not only supports cancer progression and metastasis but also impairs the function of CD8+ T lymphocytes, which are essential for anti-cancer immunity [18]. Moreover, the formation of neutrophil extracellular traps (NETs) can contribute to chemoresistance and further promote metastasis [19]. Finally, the molecular mechanisms underlying chemoresistance to drugs commonly used in metastatic cancer treatment are discussed. Relevant articles were identified through a PubMed search using keywords such as cancer, metastasis, chemoresistance, metabolism, molecular mechanisms, and immunity.

## 2. Genomics of Metastatic Cancer

**Figure 1:**
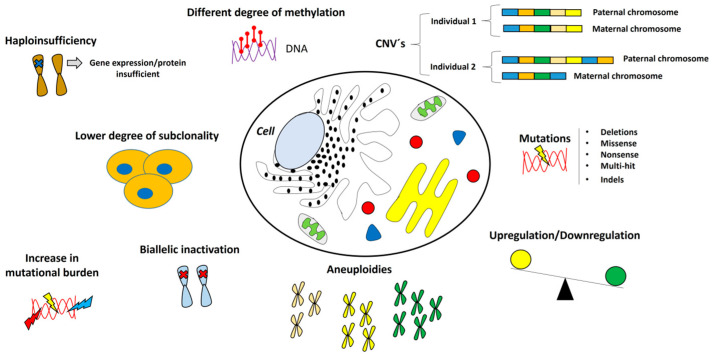
Genomic characteristics of the metastatic cell. Metastatic cells exhibit molecular, chromosomal, and mutational alterations distinct from those in primary tumors. Notably, chromosomal instability, triploid and tetraploid karyotypes, and copy number variations (CNVs) are prominent features of metastatic cells. An increase in mutational burden and mutations in tumor suppressor genes are also characteristic of metastatic tumors.

Currently, the majority of cancer-related deaths are attributed to metastases [20] and premetastatic niche (PMN) formation in distant organs is a critical step in the metastatic cascade. These niches are created through various systemic effects, such as the disruption of local fibroblasts, the recruitment of bone marrow-derived cells, and the secretion of macrophage migration inhibitory factor (MIF), which together foster a favorable environment for tumor cell invasion and spread [21,22,23,24]. Consequently, a secondary tumor may form with a mutation pattern distinct from that of the primary tumor. Interestingly, tumor progression is often more limited in metastatic tumors, which exhibit a lower degree of subclonality compared to primary tumors [6,25,26]. Some genes also show differences in methylation status between metastatic and primary tumors, highlighting the presence of tumor heterogeneity [27]. In this context, recent studies utilizing whole genome sequencing have revealed genomic profiles with elevated mutation rates in tumor suppressor genes (TSGs) in metastatic cancer patients compared to those in the primary tumor. Among the most frequent mutations in TSGs are deletions, missense mutations, nonsense mutations, and multi-hit events. Additionally, a high percentage of biallelic inactivation has been observed in genes such as *TP53*, *CDKN2A*, *RB1*, and *PTEN*, which results from mutations in both alleles, epigenetic mechanisms like promoter region methylation, and haploinsufficiency [9,25,28,29]. Metastatic tumors tend to exhibit a lower degree of subclonality and slower progression compared to primary tumors. Furthermore, point mutations, a loss of heterozygosity, and insertions/deletions (indels) are commonly found in genes involved in DNA repair, such as *BRCA1* and *BRCA2*, in samples from patients with metastatic cancer [30,31]. Kabbarah et al. (2010) observed significant chromosomal alterations in advanced melanomas compared to primary tumors, noting that CNAs are both common and more pronounced in metastatic melanoma [32]. This phenomenon is likely a driver of metastasis in other types of neoplasms as well. For instance, previous studies have shown that certain DNA segments linked to the *MYC* and *MDM4* genes are more frequently amplified in breast cancer metastases [33]. They also found a gain in the number of copies of the *MCL-1* gene on chromosome 1 in metastatic papillary thyroid cancer cells. However, they also noted a loss of the *CDKN2A* gene on chromosome 9, suggesting the presence of additional chromosomal alterations [34]. In this regard, previous studies have highlighted chromosomal instability (CIN) as a hallmark of metastasis in certain cancer types. Unlike primary tumors, metastatic cells often exhibit triploid (3n) and tetraploid (4n) karyotypes, which are linked to resistance to apoptosis and the inactivation of RB1 and p53 proteins [35]. In metastatic human cancers, mutational load and aneuploidies are typically more pronounced [20]. Additionally, CIN promotes mesenchymal features, significantly reduces survival in experimental mouse models, and has been associated with the generation of cytosolic DNA and a higher prevalence of micronuclei in CIN-high and metastasis-derived cells [36,37]. In metastatic breast cancer, an increased mutational burden and a higher frequency of genetic alterations in genes such as *TP53*, *ESR1*, *GATA3*, *KMT2C*, *NCOR1*, *AKT1*, *NF1*, *RIC8A*, and *RB1* have been observed compared to primary tumors [38]. Other studies have demonstrated that differential gene expression (DEG) is a key characteristic of metastatic progression. A distinctive phenomenon is observed between primary and metastatic melanoma tumors, where certain genes, such as *PSPH*, *SPP1*, and *IGF2BP3*, exhibit positive regulation and are preferentially expressed in the clinical samples of metastatic melanoma compared to primary tumors [18] (Figure 1). Moreover, whole exome sequencing (WES) studies have revealed the presence of clonal driver mutations in metastatic lesions following prior cytotoxic treatment. In addition, an increased prevalence of monoclonal subpopulations was observed in metastatic tumors of the colon, lung, and breast. However, in untreated metastases, it has been observed that driver gene heterogeneity is minimal [39].

## 3. Metabolic Plasticity in Cancer Metastasis

Metabolic flexibility is a key trait that enables metastatic cells to adapt to the diverse environments of different organs. It has been observed that metastatic lesions can reprogram their energy metabolism, allowing them to take up and degrade glucose even in the presence of oxygen [40]. Additionally, some cancer types exhibit distinct metabolic and enzymatic microenvironments compared to their primary counterparts [41]. For instance, Hicks et al. demonstrated that metastatic breast cancer cells have higher glycogen content compared to those from primary tumors. This may be explained by the increased activity of the enzyme phosphoenolpyruvate carboxylase (PCK), which converts oxaloacetate to phosphoenolpyruvate, initiating glucose synthesis. Furthermore, other carbon sources from the Krebs cycle can serve as precursors for glucose production, thus ensuring an energy reserve in metastatic lesions [42]. In contrast, the subpopulations of metastatic cervical cancer (CCa) cells show minimal dependence on glucose uptake and degradation. Instead, one of the primary sources of energy for these cells is the oxidative capacity of fatty acids (FAO), such as palmitic acid (PA), along with an increase in acetyl-CoA levels in metastatic lymph node (LN) cells [43]. Notably, palmitic acid has been shown to induce prometastatic memory and promote metastasis in PA-fed mice [44]. The oxidation of palmitic acid relies on mitochondrial enzymes that facilitate the transport of fatty acids into the mitochondria, including carnitine palmitoyltransferase I (CPT1), a critical effector of FAO [45]. Additionally, the extracellular lipid storage capacity within the cell plays a role in this process [17]. Previous studies have demonstrated that CPT1 is crucial for the metastatic spread and propagation of triple-negative breast cancer (TNBC) [46], and abnormal increases in CPT1 activity and FAO have been observed in gastric cancer with lymph node metastasis [16]. However, the metabolic flexibility of cancer cells enables them to adapt to various energy demands, particularly through the oxidation of certain amino acids such as glutamine. Glutamine is metabolized into glutamate by the enzyme glutaminase 1, and then glutamate is converted into alpha-ketoglutarate by glutamate dehydrogenase (GDH), which can be incorporated into the Krebs cycle [13,18]. In fact, increased glutamine oxidation has been observed in advanced melanoma, as well as elevated plasma concentrations of glutamate in patients with advanced squamous cell carcinoma of the esophagus, particularly in those with lymph node metastasis [47,48]. Additionally, intermediates from the pentose phosphate pathway, such as glucose 6-phosphate dehydrogenase (G6PD) and 6-phosphogluconate dehydrogenase (PGD), are found to be more abundant in metastases compared to subcutaneous primary melanomas [49].

Further research has explored the role of bile acids and their association with FAO signaling pathways. The accumulation of bile acids in metastatic lymph nodes is thought to activate the yes-associated protein (YAP), promoting fatty acid oxidation [50]. Moreover, elevated pyruvate levels have been described in the metastatic lung niches of breast cancer, where pyruvate serves as a precursor for the synthesis of α-ketoglutarate. This metabolite activates the enzyme collagen prolyl-4-hydroxylase (P4HA), which is involved in collagen hydroxylation, thereby influencing the extracellular matrix to favor the metastatic microenvironment in murine models [51].

On the other hand, clinical studies have highlighted metabolic acidosis in patients with metastatic esophageal cancer due to the excess lactic acid produced by tumors [52,53]. Elevated lactate levels have also been observed in the circulation of patients with colorectal cancer metastases [54], alongside significant increases in lactic dehydrogenase levels in advanced melanoma, which correlate with the number of metastatic lesions [55,56]. It is possible that the aforementioned metabolic alterations may contribute to the tumor cells’ ability to evade the effects of immunotherapy and certain alkylating drugs, such as 3-bromopyruvate (3BP). Additionally, in metastatic breast cancer, an increase in the expression of the *SHMT2* gene was observed. This gene encodes the mitochondrial form of serine hydroxymethyltransferase 2, an enzyme that catalyzes the reversible conversion of serine and tetrahydrofolate into glycine and 5,10-methylene tetrahydrofolate [41]. Other findings in the liver metastases of colorectal cancer, have documented higher levels of hypoxia-inducible factor-1α (HIF-1α) expression compared with the primary tumor, correlating with a poor prognosis [57].

Additionally, in murine models, metastatic lung and liver cancer cells have shown resistance to paclitaxel and doxorubicin. This resistance is thought to be mediated by the initial hypoxia in the primary tumor microenvironment, which can activate ABC family genes linked to drug efflux and autophagy, contributing to the evasion of chemotherapeutic agents [58]. In addition, other research groups have reported the increased mRNA expression of *CPT1B*, *STAT3*, and *ACADM* genes in biopsies from patients with treatment-resistant TNBC, as well as in paclitaxel-resistant cell lines. These findings suggest that the CPT1B–STAT3-mediated fatty acid oxidation pathway plays a crucial role in drug resistance, as metastatic cells regain sensitivity when the FAO pathway is blocked or leptin levels are reduced. Furthermore, higher levels of FAO-related metabolites, such as acylcarnitines, glutarylcarnitine, and propionylcarnitine, have been reported in chemoresistant cells [59].

**Figure 2:**
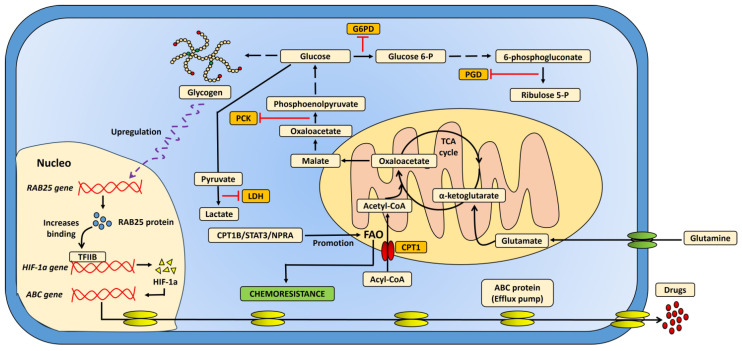
The metabolic reprogramming of metastatic tumors and its influence on chemoresistance. Metabolic flexibility enables metastatic cells to utilize various energy sources, which is crucial for their survival and progression. Glutamine degradation contributes to the Krebs cycle by incorporating substrates through glutamate, while increased fatty acid oxidation promotes both chemoresistance and the production of acetyl-CoA. These non-glycolytic pathways supply essential energy to metastatic cells, facilitate glucose synthesis, and activate the pentose phosphate pathway. Additionally, gluconeogenesis and glycogen storage can trigger molecular signaling that stimulates HIF-1α synthesis. The expulsion of chemotherapy drugs is mediated by the expression of efflux pumps, such as ABC proteins, which help the cells evade treatment. The orange boxes in the figure represent enzymes that are upregulated in metastatic cancer, highlighting their role in supporting chemoresistance in these cells.

Recent evidence suggests that the natriuretic peptide A receptor (NPRA) may promote FAO and be linked to cisplatin resistance in gastric cancer treatment [60]. Furthermore, previous studies have shown that in murine models, mitochondrial function is reduced in metastases compared to primary tumors. Metabolic flexibility allows metastatic cells to utilize different substrates for energy production, enhancing their adaptability [54]. Additionally, the growing body of evidence supports the potential to establish metabolic models that are associated with drug resistance (Figure 2).

## 4. Metastatic Cancer and Drug Resistance

The treatment of metastatic cancer faces significant challenges, particularly in the context of drug resistance, which complicates the effectiveness of current chemotherapy and immunotherapy approaches [61]. Genetic variations play a critical role in the therapeutic failure observed in metastatic tumors. For example, in estrogen receptor-positive (ER+) metastatic breast cancer, missense mutations in the *ESR1* gene have been linked to a lack of pharmacological response to selective estrogen receptor modulators (SERMs) and endocrine therapy [62]. Additionally, exon translocation events in the *ESR1* gene such as those involving *CCDC170*, *YAP1*, and *SOX-9* lead to the production of fusion proteins that reduce sensitivity to drugs like tamoxifen and fulvestrant [63,64]. As a result, estrogen receptor (ER) activation can occur even in the absence of the ligand, despite the presence of endocrine inhibitors used in the treatment of metastatic breast cancer. This process is often associated with the constitutive activation of the PI3K/AKT and MAPK signaling pathways, which contribute to drug resistance [65].

Furthermore, mutations in the *ESR1* gene can induce conformational changes in the receptor, altering its affinity for SERMs and complicating treatment outcomes [62]. In addition to genetic alterations, increases in the expression of efflux transporters, such as P-glycoprotein (P-gp), have been observed in the ectopic models of human metastatic neuroblastoma [66]. P-gp, a glycoprotein membrane transporter, can limit the absorption and bioavailability of drugs like imatinib, thereby reducing their efficacy in treating certain cancers, such as chronic myeloid leukemia [67]. However, drug resistance mechanisms in metastatic cancer extend beyond the cellular expulsion of antineoplastic drugs. Therapeutic failure is also common with cyclin-dependent kinase inhibitors (CDK4/6 inhibitors) in the treatment of metastatic breast cancer [68]. Additionally, the participation of cancer stem cells (CSCs) in therapeutic resistance is well-documented. These cells possess the ability to self-renew and differentiate, which contributes to their persistence and resistance to treatment [69,70].

In murine models, metastatic cells with low tumor burden or those originating from tissues in the early stages of metastasis have been shown to express stem cell-associated genes such as *LGR5*, *BMI1*, *NOTCH4*, and *JAG1*. These cells differ significantly from the primary tumor cells from which they originated [71]. This finding supports the hypothesis that metastatic disease is closely associated with the presence of cancer stem cells, which possess the capacity to self-renew and differentiate, thus driving cancer recurrence [72]. Notably, these stem-like cells can persist even after chemotherapy, as the DNA of tumor cells is susceptible to “de novo” mutations and clonal driver mutations induced by cytotoxic treatment [39,73].

Additionally, an imbalance in the expression of antiapoptotic proteins has been implicated in metastatic cancer. For instance, the overexpression of Bcl-2 in tumor tissues from patients with advanced prostate cancer correlated with higher Gleason scores [74]. Moreover, chemoresistance has been linked to the epithelial-mesenchymal transition (EMT), a critical process that occurs prior to cancer invasion and metastasis. EMT is a complex event involving various molecules, such as 14,15 EET, USP37, CCL5, and NLRP3. Abnormal expression of these molecules during EMT can reduce sensitivity to conventional chemotherapies like cisplatin, epirubicin, gemcitabine, and tamoxifen [75].

Therapeutic failure is also observed with immunotherapies targeting immune checkpoints, including anti-PD-1, anti-PD-L1, and anti-CTLA-4 antibodies, as well as epidermal growth factor receptor (EGFR) inhibitors like cetuximab and panitumumab. Resistance to these therapies is often linked to specific molecular characteristics in patients with metastatic cancer (Table 1), such as the absence of microsatellite instability, PD-L1 negativity, low tumor purity, and inadequate immune infiltration [76,77]. Additionally, resistance to cetuximab and panitumumab in metastatic colorectal cancer (mCRC) has been associated with the increased expression of EGFR ligands, structural alterations in EGFR, and autophagy [78]. Other findings have demonstrated primary and acquired resistance to immune checkpoint inhibitors (ICIs) during cancer treatment [79]. In primary resistance, individuals with a high total tumor mutational load (TML) have been observed to show an unfavorable response to immunotherapy. Furthermore, the induced death of natural killer cells (NK), CD8+ T cells, and B lymphocytes, as well as the infiltration of FoxP3+ Tregs in tumor tissue, can be predictive markers of primary resistance to ICIs. However, the expression of immunosuppressive cytokines by Tregs, such as IL12A, TGFβ, IL-10, and IL-35, has also been associated with a poor prognosis [80,81]. On the other hand, acquired resistance to immunotherapy can be triggered by multiple causes. For example, mutations in the *B2M* gene could lead to the loss of MHC class I and defects in antigen presentation. Furthermore, the loss of the tumor suppressor *PTEN*, as well as alterations in the JAK/STAT signaling pathway and defects in IFN-γ response, are some of the most relevant mechanisms [82].

Other studies have demonstrated that biophysical changes in the extracellular matrix and increased lipolytic activity in the hepatic tumor microenvironment contribute to the lack of response to antiangiogenic therapy with bevacizumab in mCRC patients [91]. However, these effects can be reversed by reducing tissue stiffness and inhibiting fibroblast contraction, which is often associated with metastases [92].

Finally, in advanced melanoma, a significant portion of patients develop resistance to anti-PD-1 inhibitors. Nevertheless, clinical responses improved when anti-PD-1 therapy was combined with ipilimumab and nivolumab [85,86]. In contrast, patients with colorectal cancer who develop liver metastases often show resistance to immunotherapy, even when combined with regorafenib and nivolumab, or regorafenib with both ipilimumab and nivolumab. These findings suggest that the site of metastatic disease plays a critical role in determining the prognosis and pharmacological resistance to such therapies [83].

## 5. Immunity and Chemoresistance in Metastatic Cancer

NK cell activity, along with the activation of CD8+ lymphocytes, play a key role in tumor suppression [97,98]. However, even in immunologically competent individuals, some cancers can develop and progress [99]. Understanding the influence of the immune microenvironment on metastatic lesions, particularly its relationship with chemoresistance, is therefore critical. Previous studies have shown a reduction in the expression of T and B lymphocytes, as well as NK cells and fibroblasts, in TNBC metastases. This reduction likely inhibits the immune response against tumors [33]. Moreover, cancer-associated fibroblast infiltration, and the recruitment of regulatory T cells (Tregs) and neutrophils driven by the tumor microbiome, have been linked to immunosuppression in advanced cancer [100]. Specifically, Tregs have been observed to play an important role in resistance to ICIs. One possible mechanism that may explain this phenomenon is the increase in their effector function, driven by the binding of coinhibitory receptors CTLA-4 and PD-1, expressed on Tregs, to their respective ligands on tumor cells. This binding also promotes the development of an immunosuppressive microenvironment and a diminished antitumor response [80]. Additionally, cytotoxic treatments are believed to promote the recruitment of segmented leukocytes and resistance to cisplatin and cyclophosphamide in murine models with lung metastasis through the formation of NETs composed of DNA strands, histones, inflammatory mediators, and proteins released by neutrophils [101,102,103]. Other findings have indicated that certain antioxidant enzymes, such as NAD(P)H:quinone oxidoreductase (NQO1), play a role in promoting NET formation, as well as tumor progression and lung metastasis. This occurs through the upregulation of peptidyl-prolyl cis-trans isomerase A (PPIA) [104]. Additionally, recent evidence suggests that chemoresistant and ferroptotic neutrophils from patients with aggressive breast cancer, who have been treated with taxanes and cyclophosphamide, can suppress CD8+ T cell proliferation and the production of effector enzymes like perforins and granzyme B [105]. This suppression may be linked to a decreased capacity of CD8+ T cells to infiltrate the tumor site, thereby contributing to therapeutic resistance [52,106]. Interestingly, previous studies have also demonstrated that inhibiting NET production in the metastatic microenvironment enhances CD8+ T cell activity [19].

**Figure 3:**
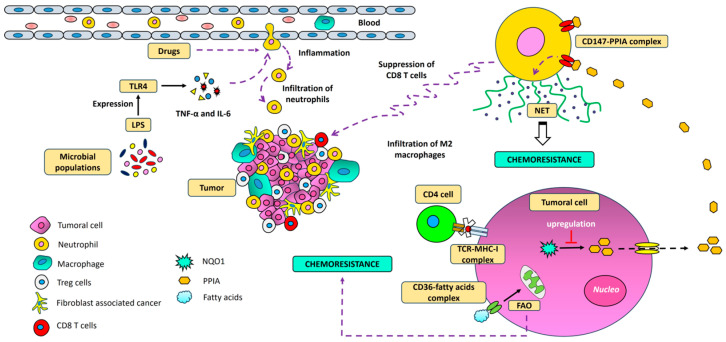
The influence of neutrophils on the metastatic microenvironment. Neutrophil infiltration into tumors can be influenced by microbial factors within the tumor microenvironment. Neutrophils that exhibit resistance to cytotoxic therapies have been shown to promote the formation of NETs through the activation of peptidyl–prolyl cis–trans isomerase A (PPIA). These NETs, in turn, suppress the activation of CD8+ T lymphocytes and contribute to chemoresistance. Additionally, FAO can drive macrophage infiltration, further enhancing resistance to cancer therapies. Furthermore, NAD(P)H:quinone oxidoreductase 1 (NQO1) has been implicated in these processes, influencing both immune responses and therapeutic efficacy in the tumor microenvironment.

On a different note, certain surface markers expressed by tumor cells, such as CD36, have been associated with metastasis, monocyte infiltration, and the subsequent differentiation of these monocytes into pro-tumor M2 macrophages. These macrophages secrete cytokines that promote inflammation and chemotaxis [107,108]. CD36 is a transmembrane glycoprotein with a high affinity for fatty acids like palmitic acid and functions as an important receptor for dietary lipids [109]. In metastatic liver cancer and oral carcinoma, CD36 expression is notably higher than in non-metastatic tumor cells [108,110]. In chronic myeloid leukemia, CD36 can enhance blast migration, drive metastasis, and contribute to relapse after cytotoxic treatment [111]. Fatty acid metabolism, in this context, is emerging as a hallmark of metastatic cells and chemoresistance (Figure 3). However, therapeutic failure in metastatic cancer can stem from multiple factors, including alterations in the presentation of tumor antigens through major histocompatibility complex (MHC). Clinical studies have demonstrated that individuals with a higher expression of MHC-I and MHC-II show a better response to drugs like eribulin [77]. In vitro studies have also shown that metastatic lung cells in murine models express minimal amounts of MHC-I, leading to the incomplete presentation of tumor antigens and the loss of neoantigens [112]. Additionally, in breast cancer metastases, a significant downregulation of MHC-I-related genes, including *HLA-A*, *HLA-B*, and *HLA-C*, has been observed [33].

Additionally, circulating tumor cells (CTCs) that escape the primary tumor and contribute to metastasis express transforming growth factor-β receptor type 1 (TGFβ-RI), which facilitates the formation of platelet–CTC clusters. This complex prevents CTCs from being destroyed by NK cells and promotes immune evasion [113]. Other findings have described that CD47, a surface marker present on tumor cells, can inhibit effector phagocyte-mediated prophagocytosis signals [114], and in some metastatic cancers, significant increases in its expression have been observed. For example, in metastatic brain tumors, an 89% increase in CD47 expression has been demonstrated compared to normal adjacent tissue in biopsies from patients with that type of cancer [115].

Finally, it is important to consider the relationship between the immune system and tumor latency because metastatic relapses can occur in cancer patients months after the initial diagnosis and treatment. In this regard, it has been observed that tumor cells have the ability to survive and reactivate their proliferation cycle in host organs when they evade innate immunity cells, such as NK cells, by downregulating death inducing receptors [116].

## 6. Conclusions

Metastatic lesions exhibit molecular behaviors that are distinct from those of the primary tumor. As tumor cells acquire a metastatic phenotype, they develop unique features such as reduced subclonality and chromosomal aberrations, often presenting with triploid or tetraploid karyotypes. Secondary tumors commonly display additional genetic alterations, including CNVs, an increased mutational burden, and the biallelic inactivation of tumor suppressor genes such as *TP53* and *RB1*. Metastatic cells also demonstrate remarkable metabolic flexibility, enabling them to adapt to diverse microenvironments by reprogramming their energy metabolism. This includes promoting gluconeogenesis through the degradation of fatty acids, glutamine, and other carbon sources from the Krebs cycle. The produced glucose fuels energy demands and supports glycogen synthesis, which in turn can enhance the expression of HIF-1α and ATP-binding cassette (ABC) transporters, both of which contribute to chemoresistance. Additionally, the formation of NETs plays a critical role in mediating drug resistance, suppressing CD8+ T cell activity, and promoting a pro-inflammatory tumor microenvironment. During EMT, the overexpression of factors such as USP37, CCL5, and NLRP3 has been shown to diminish the efficacy of anticancer therapies and alter drug sensitivity. Structural variations in receptors targeted by therapeutic antibodies, along with biophysical changes in the extracellular matrix, have been linked to resistance against antiangiogenic treatments. Moreover, deregulation of key signaling pathways—including PI3K–AKT, RB–E2F, and MEK—as well as abnormal PD-L1 expression, are frequently observed in metastatic tumors and are associated with resistance to ICIs.

## Figures and Tables

**Table 1:** Drugs associated with drug resistance in metastatic cancer.

Drug Family		Metastasis Associated with	Mechanisms Associated with Resistance	Reference
**Endocrine therapy**				
Tamoxifen Fulvestrant		Breast cancer	Alterations in the *ESR1* gene, including the following: Loss, amplification, translocation; Decreased proteolytic degradation; Decreased affinity to drugs. Alterations in the PI3K–AKT–mTORC1, RAS–MAPK, and CDK4/6-RB–E2F pathways	[62]
			Translocation of exons in the *ESR1 CCDC170*, *YAP1*, and *SOX-9* genes	[63,64]
**ICIs**				
Anti-PD-1	Nivolumab	Colorectal cancer	-----------------------	[83]
		Melanoma	Alteration in PD-L1 expression	[84]
		Colorectal cancer	Microsatellite instability status	[76]
	Pembrolizumab	Breast cancer	The downregulation of PD-L1, tumor purity, lower levels of immune infiltration, estrogen signaling	[77]
		Melanoma	Upregulation of genes like *TIM3* and *CTLA-4*	[85]
Anti-PDL-1	Atezolizumab	------------	BRAF mutation Overactivation of MEK pathway	[86]
Anti-CTLA-4	Ipilimumab	Melanoma	The secretion of IL-6, IL-10, and TGF-beta cytokines; high levels of CD4+, CD25+ FoxP3+, and Tregs; high expression of IDO and PDL-1; loss of the *PTEN* gene, mutations in JAK1 and JAK2; the activa tion of WNT/beta-catenin pathway	[84,87]
**CDK4/6i**				
Palbociclib		Breast cancer	Overexpression of the *CCNE1* gene	[88]
			High expression of cyclin E mRNA	[89]
			The downregulation of Rb1, up-regulation in the G2-M pathway, the upregulation of STAT3 and EMT by IL6	[90]
			The loss of Rb1, alterations in the *AKT1*, *ERBB2*, and *FGFR2* genes, amplification of the *AURKA* and *CCNE2* genes	[68]
Abemaciclib		Breast cancer	The high expression of oxidative phosphorylation genes (*OXPHOS*) Reactive oxygen species (ROS)	[90]
**Anti-VEGF**				
Bevacizumab		Colorectal cancer	Matrix stiffness, fatty acid oxidation, angiogenesis	[91,92]
**Anti-EGFR**				
Cetuximab Panitumumab		Colorectal cancer	Overexpression of the *EGRF* gene, structural alterations in EGRF, autophagy, metabolic remodeling, microsatellite instability, alteration of the tumor microenvironment, angiogenesis	[78]
**TKI’s**				
Imatinib		Chronic myeloid leukemia	Overexpression of the efflux pumps (P-gp)	[67]
**Others**				
Eribulin		Breast cancer	Tumor heterogeneity, estrogen signaling, lower antigen presentation	[77]
Cisplatin		Colorectal cancer	ABCG2 efflux pump, secretion through exosomes	[93]
		Gastric cancer	The overexpression of miR-21/aberrant expression of miRNAs	[94,95]
		Breast cancer	The overexpression of 14,15 EET and USP37, integrin αvβ3 expression	[75]
Epirubicin		Breast cancer	Plasma levels of CCL5	
Paclitaxel		Gastric cancer Lung/Liver	Downregulation of the CDH11 protein and dysregulated alfa-1β protein Hypoxia/the activation of *ABC* genes/autophagy	[58,96]
